# Prevalence of inappropriate antibiotic prescriptions after the great east Japan earthquake, 2011

**DOI:** 10.1097/MD.0000000000006625

**Published:** 2017-04-14

**Authors:** Kentaro Iwata, Takahiko Fukuchi, Midori Hirai, Kenichi Yoshimura, Yasuhiro Kanatani

**Affiliations:** a Division of Infectious Diseases Therapeutics, Kobe University Graduate School of Medicine, Kobe; b Department of General Medicine, Saitama Medical Center Jichi Medical University, Saitama; c Department of Pharmacokinetics and Pharmaceutics, Kobe University Graduate School of Medicine, Kobe; d Innovative Clinical Research Center (iCREK), Kanazawa University Hospital, Kanazawa; e Department of Health Crisis Management, National Institute of Public Health, Wako, Japan.

**Keywords:** antibiotic prescriptions, antimicrobial stewardship, disaster management, the Great East Japan Earthquake

## Abstract

Few studies have investigated the appropriateness of antibiotic use in postdisaster settings. We retrospectively evaluated clinical databases on health care delivered at clinics near shelters set up after the Great East Japan Earthquake, 2011. We defined appropriate, acceptable, and inappropriate antibiotic use for each diagnostic category, by applying and adopting precedent studies and clinical guidelines. From March to July, 2011, a total of 23,704 clinic visits occurred at 98 shelters with 7934 residents. Oral antibiotics were prescribed a total of 2253 times. The median age of the patients was 48.5 years old (range 0–97), and 43.7% were male. Of 2253 antibiotic prescriptions, 1944 were judged to be inappropriate (86.3% 95% CI 84.8%–87.7%). The most prescribed antibiotic was clarithromycin (646 times, 28.7%), followed by cefcapene pivoxil (644 times, 28.6%), levofloxacin (380, 16.9%), cefdinir (194, 8.6%), and cefditren pivoxil (98, 4.4%). The most frequent diagnosis for which antibiotics were prescribed was upper respiratory infection (URI, 1040 visits, 46.2%), followed by acute bronchitis (369, 16.4%), pharyngitis (298, 13.2%), traumatic injuries (194, 8.6%), acute gastroenteritis (136, 6.0%), urinary tract infections (UTIs, 123, 5.5%), and allergic rhinitis (5.1%). The majority of antibiotics prescribed at clinics after the Great East Japan Earthquake was inappropriate. Significant improvement of the use of antibiotics in postdisaster settings should be sought immediately in Japan.

## 1 Introduction

Natural disasters often result in the mass evacuation of people to shelters.^[1]^ Postdisaster disease trends may differ from one disaster to another, but many involve communicable diseases.^[2–6]^ Antibiotics had been frequently prescribed in these settings. Studies had evaluated the appropriateness of antibiotic use in regular settings,^[7–9]^ but ones for healthcare in postdisaster settings have not been evaluated.

The Tohoku area in Japan was hit by a massive earthquake with a magnitude of 9.0 and a subsequent tsunami on March 11, 2011, which resulted in more than 15,000 deaths (the Great East Earthquake).^[10]^ The earthquake also led to mass evacuation with many people staying in shelters. To evaluate the appropriateness of antibiotic use there, we reviewed clinical data on the medical services provided at these shelters.

## 2 Methods

### 2.1 Setting and patients

We extracted clinical data from database at Department of Health Crisis Management, National Institute of Public Health. The database was produced based on data from clinical charts used to provide care at 98 shelters or their accessory aid stations at Ishinomaki City, Miyagi, Japan. Ishinomaki was one of the most damaged areas following the Great East Japan Earthquake, mostly due to the massive tsunami.^[10]^ More than 50,000 people were evacuated to shelters following the tsunami, and more than 3800 people died or went missing.^[11]^ Japanese Red Cross Ishinomaki Hospital was the only designated disaster hospital in the Ishinomaki Medical Zone, and it was undamaged by the earthquake. The Ishinomaki Zone Joint Relief Team coordinated medical support from all over Japan, and medical workers provided care to people in shelters, typically rotating weekly.^[12]^ Many shelters and their aid stations lacked lifelines such as electricity, water, or sewerage systems, and medical care was provided without laboratory tests or imaging studies. Those who needed referral were transferred to Japanese Red Cross Ishinomaki Hospital, but transfer was difficult due to destroyed roads and lack of vehicles. The study was approved by the ethic committees both at Kobe University Graduate School of Medicine and Japanese Red Cross Ishinomaki Hospital.

### 2.2 Estimating appropriate antibiotic use

We included patients who were prescribed oral antibiotics at each clinic. The use of intravenous, intramuscular, or topical antibiotics were excluded from the analyses. We collected age, sex, diagnosis, and prescriptions from the database, and evaluated the appropriateness of antibiotic use.

For the definition of appropriateness, we applied and adapted the criteria developed by Fleming-Dutra et al,^[9]^ which were based on clinical guidelines for appropriate antibiotics prescription by age group in the outpatient setting. For example, antibiotic therapy was not recommended for upper respiratory infections (URI) for both adults and children in this criteria.^[13–15]^ We judged the criteria were useful and applicable in the postdisaster setting in Japan.

For acute gastroenteritis, which was the 2nd most common infectious disease after URI in a precedent study but no recommendation was made by Fleming-Dutra et al,^[16]^ we applied and adapted the Japanese Association for Infectious Diseases (JAID) and Japanese Society of Chemotherapy (JSC) guideline as the reference, which recommended no antibiotic therapy for mild acute gastroenteritis in children, but recommended clarithromycin, amoxicillin, fosfomycin, or norfloxacin for suspected *Campylobacter* infections.^[17]^ Although meta-analysis on antibiotic treatment for *Campylobacter* infection showed only limited effects,^[18]^ we allowed this antibiotic use for this condition, considering the significant damage to quality of life at shelters. We also accepted other fluoroquinolones such as levofloxacin as an alternative to norfloxacin being mindful of the possible lack of stock at each clinic. For muscloskeletal traumatic injuries, we applied Lane et al^[19]^ as a reference for recommendation, but allowed other antibiotics such as amoxicillin/clavulanate or even the use of fluoroquiolones for the same reason above. For skin and soft tissue infections (SSTI) such as cellulitis, we used the latest Infectious Diseases Society of America guideline.^[20]^

We categorized the use of antibiotics to appropriate, acceptable, or inappropriate. Acceptable antibiotic use was defined as the use of an antibiotic which was not necessarily ideal, but which can be used as an alternative to the first choice, particularly in disaster situations. For example, the use of fluoroquinolones for SSTI cannot be recommended routinely due to their broad spectrum nature, but may be acceptable in circumstances where no other antibiotics were available. We restricted the recommended antibiotics to those approved in Japan. We considered oral 3rd-generation cephalosporins not appropriate for use in most circumstances, due to poor bioavailability, their broad spectrum, and the existence of better alternatives.^[21]^ Likewise, we did not include fosfomycin in our recommendation since the fosfomycin calcium which is available in Japan has much worse bioavailability than the fosfomycin tromethamine available abroad.^[22]^ For other miscellaneous infections, we used our expert recommendations as the basis for the appropriateness of the use of antibiotics. On the other hand, we allowed the use of cefaclor for the treatment of SSTI since it is usually regarded as “first-generation cephalosporin” in Japan, instead of 2nd-generation in other countries. Cefaclor has reasonable bioavailability,^[23]^ and we judged that it can be used as an alternative to cephalexin for the management of SSTIs. If there is an ambiguity between appropriate/acceptable and inappropriate, 2 infectious diseases specialists discussed the issue to resolve the issue (KI and TF). These recommendations were summarized in Table 1.

**Table 1:**
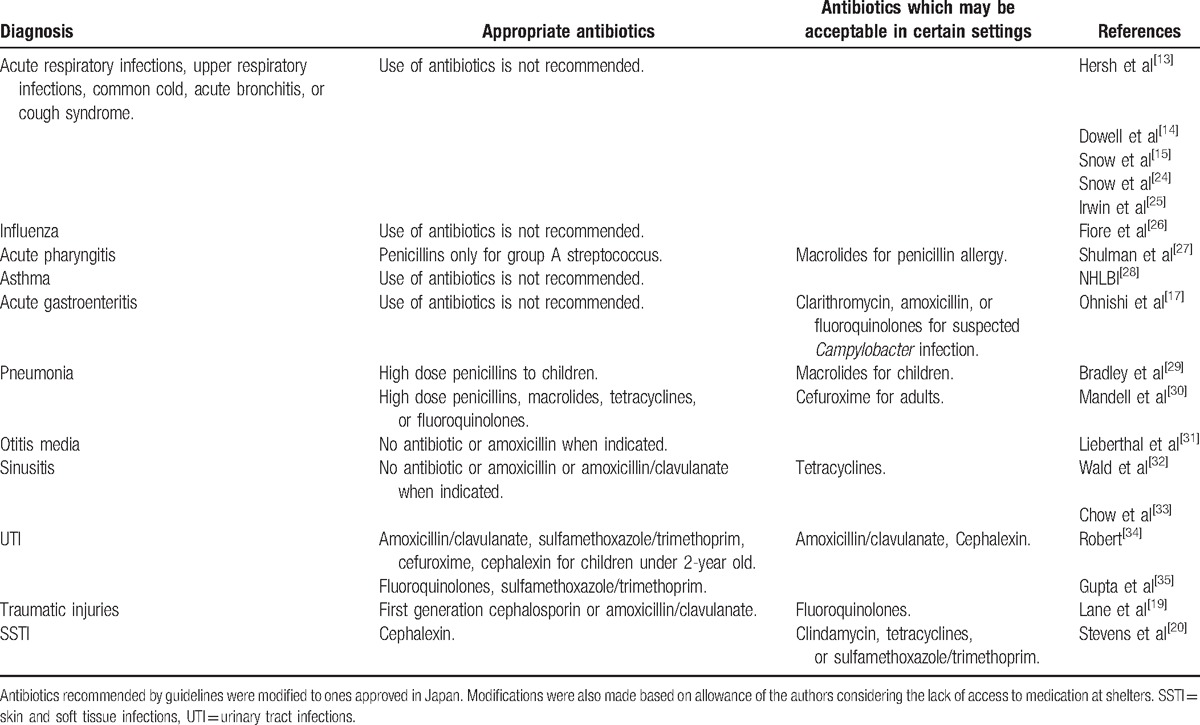
Clinical practice guideline recommendations for adults and children by diagnosis.

### 2.3 Statistical analyses

Statistical analyses were performed using STATA 14.1 (STATA Corp). Ninety-five percent confidence intervals (CIs) were calculated for estimates.

## 3 Results

From March to July, 2011, a total of 23,704 clinic visits occurred at 98 shelters with 7934 residents. Oral antibiotics were prescribed 2253 times. The median age of the patients who were given antibiotics was 48.5 years old (range 0–97), and 43.7% were male.

Of 2253 antibiotic prescriptions, 156, 153, and 1944 were judged to be appropriate, acceptable, and inappropriate, respectively (Table 2). This means that only 6.9% of antibiotic prescriptions were appropriate (95% CI 5.9%–8.1%), 6.8% were acceptable (95% CI 5.8%–7.9%), and the remaining 86.3% were inappropriate (95% CI 84.8%–87.7%).

**Table 2:**
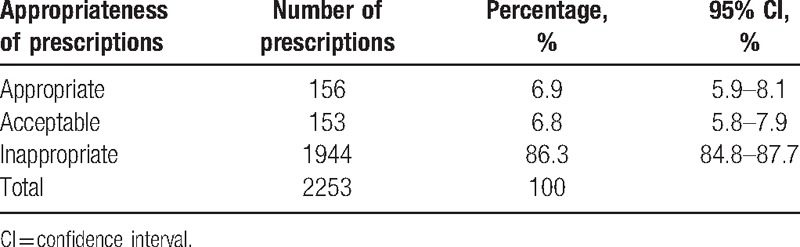
Appropriateness of antibiotics prescription at temporary clinics, Ishinomaki City, March to July, 2011.

The most prescribed antibiotic was clarithromycin (646 times, 28.7%), followed by cefcapene pivoxil (644 times, 28.6%), levofloxacin (380, 16.9%), cefdinir (194, 8.6%), and cefditren pivoxil (98, 4.4%). For each antibiotic, most prescriptions were judged to be inappropriate (Table 3).

**Table 3:**
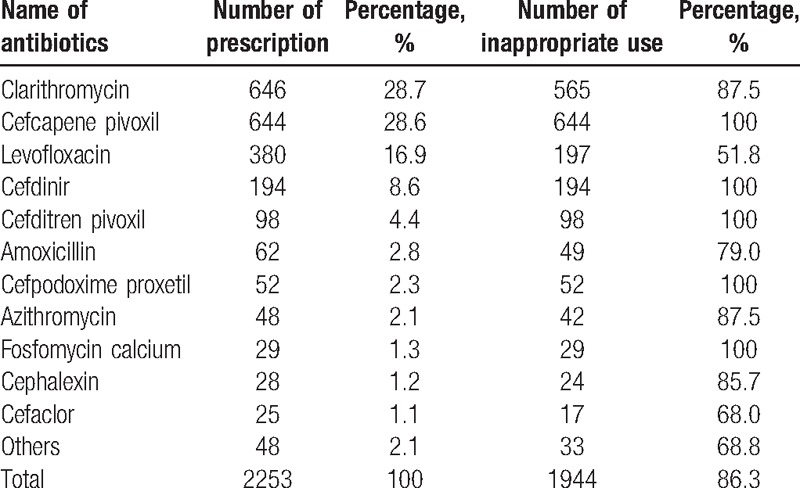
Frequency of antibiotics prescriptions and their appropriateness.

The most frequent diagnosis for which antibiotics were prescribed was URI (1,040 visits, 46.2%), followed by acute bronchitis (369, 16.4%), pharyngitis (298, 13.2%), traumatic injuries (194, 8.6%), acute gastroenteritis (136, 6.0%), urinary tract infections (123, 5.5%), and allergic rhinitis (5.1%) (Table 4). Again, antibiotics were used inappropriately in most diagnoses except for urinary tract infection and pneumonia, with inappropriate antibiotic use of 17.5% and 25.9%, respectively.

**Table 4:**
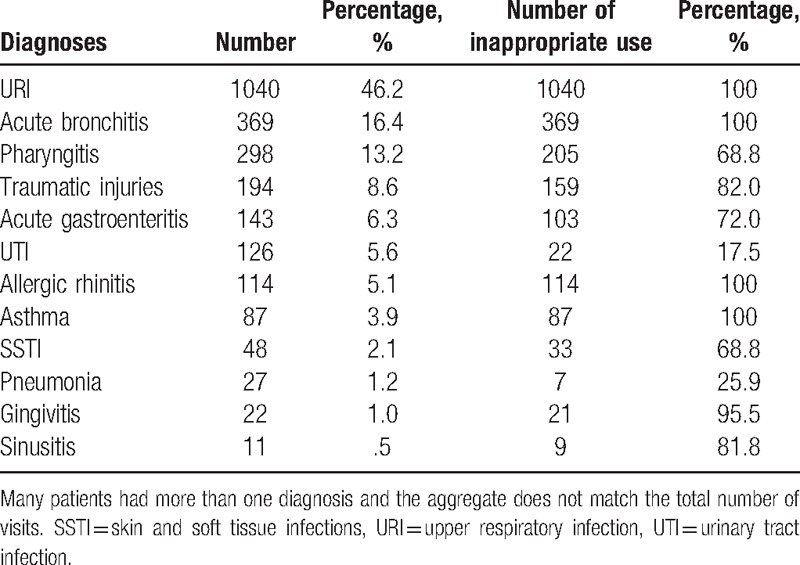
Demographics of diagnoses where antibiotics were frequently prescribed.

## 4 Discussion and conclusions

Our analyses describe the appropriateness of antibiotics used at clinics in Ishinomaki city after the Great East Japan Earthquake. We found most antibiotics were used inappropriately, and the most of them were given for diagnoses such as URI, for which no antibiotics are recommended. For other diagnoses where antibiotic use is recommended or justified, we found many cases of the misuse of antibiotics.

Fleming-Dutra et al^[9]^ estimated the prevalence of inappropriate antibiotic prescriptions among ambulatory care visits in the United States, and found that 153 out of 506 annual antibiotic prescriptions per 1000 population were not appropriate (30.2%).^[9]^ Although there are studies investigating the amount of antibiotic consumption in Japan,^[36]^ few studies have investigated their appropriateness. Our findings, even though criteria for appropriateness are slightly different, were much worse than those in the United States.

According to Muraki et al,^[36]^ although, the rate of defined daily doses per 1000 inhabitants per day for penicillin was much smaller in Japan than in European countries, oral 3rd-generation cephalosporins, oral macrolides, and oral fluoroquinolones were consumed at high rates in Japan. Our study findings are consistent with them, and we found that not only these broad spectrum antibiotics were used frequently, but also they were used inappropriately.

The overuse of 3rd-generation cephalosporins poses significant risk to society. Oral 3rd-generation cephalosporins generally have poor bioavailability, yet can kill Enterobacteriaceae in the intestine unnecessarily, which can lead to an increase in antibiotic resistant organisms and *Clostridium difficile* infection.^[22,37,38]^

In our study, the use of macrolides was accepted for the treatment of pharyngitis and acute gastroenteritis. Macrolides are recommended for acute bacterial pharyngitis when penicillin cannot be used due to allergy.^[27]^ Also, the use of macrolides were judged to be acceptable in treating selected cases of *Campylobacter* infection.^[17]^ Despite this allowance, most macrolides prescribed in our study were inappropriate. Inappropriateness might be even worse than our estimate, since most cases of pharyngitis are caused by viral infection, and many can be treated with penicillins even if caused by bacteria. Additionally, most diarrheal illnesses are not caused by *Campylobacter*. However, given the circumstances where few diagnostic tests were available, we had to accept the potential overtreatment with macrolides to a certain extent. Despite this allowance, macrolides, particularly clarithromycin, were overused and misused according to our findings.

The overuse of macrolides in Japan has resulted in an increase in macrolide resistant organisms, such as *Mycoplasma pneumoniae*, *Streptococcus pneumoniae*, and *Streptococcus pyogenes*.^[39,40]^ In addition, the use of macrolides is associated with significant side effects such as cardiovascular death.^[41]^ Clarithromycin is known to increase the risk of rhabdomyolysis when coadministered with statins.^[42]^ Even though macrolides can be useful in certain infections, they should never be used unnecessarily, particular in postdisaster settings, where one may not be able to treat the complications of medication use promptly.

We are not trying to make an assertion that the health care workers that provided care after the Great East Japan Earthquake were incompetent or ignorant. We simply sought the appropriateness of antibiotic prescriptions. The inappropriateness could come from number of factors, such as the lack of antibiotic stock at each clinic, the need to deliver hope to devastated people at shelters, or the lack of diagnostic tools to name a few. Some volunteer doctors might be specialists of different areas and might not be used to prescribe antibiotics at such settings (We have no data on demographics of specialties of volunteer doctors during this period). In Japan, not every physicians received training on primary care, not to mention the field of “disaster medicine.” We were able to reveal “what happened” after the earthquake, but were not able to reveal “why” this happened. In-depth investigations such as qualitative studies might reveal the root cause of antibiotic misuse.^[43]^

We investigated the cases where antibiotics were used, but we did not investigate the cases where antibiotics were not used. For example, 2739 diagnoses of URI were made during the study period, and antibiotics were prescribed in 1040, suggesting URI were appropriately managed in more than half the cases (62.0%). This is similar to antibiotic prescription rate for URI at nondisaster setting according to a cross-sectional study in Japan (60%).^[44]^ Further studies are needed to evaluate to what extent inappropriate antibiotic use is due to unusual environment after disasters, or due to inappropriate antibiotic use in daily practice.

There are several limitations inherent in our study. First, since we extracted data for the diagnoses and the names of the antibiotics used, we might be missing important contextual information on each practice. There might have been justifiable reasons to prescribe antibiotics in individual cases, for which we judged them inappropriate. On the other hand, since we were not able to obtain detailed information about patients, there might have been more antibiotic misuse than we identified, such as contraindications due to underlying health problems, interactions with other medications, or the presence of known allergies. Second, our criteria of appropriate, acceptable, or inappropriate antibiotic use might be seen as too austere. However, we applied precedent studies on appropriate antibiotic use and current clinical guidelines. We were also mindful of the extreme environment after disasters, allowing rather broad spectrum antibiotics for certain infections, such as fluoroquinolones for SSTIs. We are afraid that we might have been underestimating the proportion of inappropriate antibiotic use, but we do not consider that we overestimated it.

In conclusion, we found a significant number of inappropriate antibiotic use at clinics held after the Great East Japan Earthquake. Japan suffered another large scale earthquake in the Kumamoto area in 2016,^[45]^ and we will definitely experience other disasters with health care needs.

We believe that even refugees after disasters do have the right to receive appropriate medications, such as antibiotics for their health and environment. Further improvement in the use of antibiotics in such circumstances should be undertaken.

## Acknowledgments

The authors thank Dr Seiichi Kobayashi for invaluable comments and discussion. The authors also thank Dr Daniel Mosby for editing the manuscript to eliminate grammatical or spelling errors.
